# Supplementary material for: Ultrathin crystalline-silicon-based strain gauges with deep learning algorithms for silent speech interfaces
Source: Nat Commun. 2022 Oct 3;13:5815. doi: 10.1038/s41467-022-33457-9 (PMC9530138; doi:10.1038/s41467-022-33457-9)
Supplement: Supplementary file 1 — Supplementary Information [file 41467_2022_33457_MOESM1_ESM.pdf]

Supplementary Information:

Ultra-thin crystalline silicon-based strain gauges with deep learning algorithms for silent speech interfaces

Yu et al.

Supplementary Table 1. A performance comparison of silent communication systems based on strain gauges

Sensing Technique	Material	Gauge factor	Channel	Attachment position	Language (type)	Class	Accuracy	Reference
Strain	Single crystalline silicon	155	8	Face	English (verbal)	100 words	87.53%	This work
Strain	Graphene	-	1	Throat (Vocal cord)	English + motion (verbal + nonverbal)	15 words + 4 motions	55% (words) 85% (motion)	[1]
Strain	Au nanomesh	7.26 ~ 46.3	18	Face	English vowels (verbal)	3 vowels	-	[2]
Strain	Graphene-coated silk-spandex	19.6 ~ 34.3	10	Fingers	Gesture (nonverbal)	4 gestures	96.07	[3]
Strain + vision	Single-walled carbon nanotube / polydimethylsiloxane	-	5	Hand (Fingers)	Gesture (nonverbal)	10 gestures	100% (normal) 96.7% (noisy)	[4]
Strain	Carbon grease	8	5	Fingers	Sign language (nonverbal)	10 gestures	98%	[5]

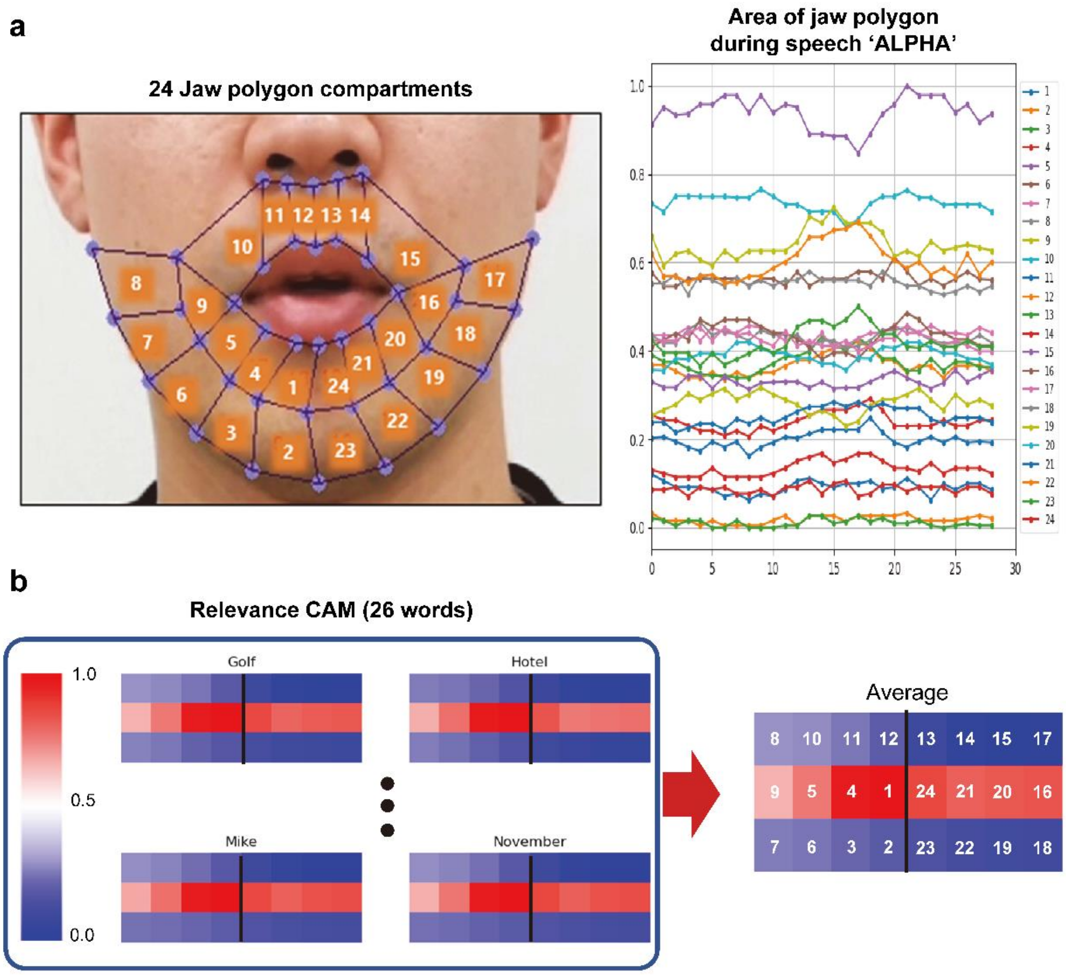

Supplementary Figure 1. Decision of sensor locations through area feature analysis (R-CAM). a, Picture of 24 randomly partitioned compartments of facial skin near the mouth (left) and area change of 24 jaw polygons in the time domain while the word "ALPHA" is silently spoken (right). **b,** R-CAM analysis of 26 NATO words, indicating which compartments have significant areal change. It was confirmed that the areal change of the sections under the lower lip was the most remarkable while speaking the words silently.

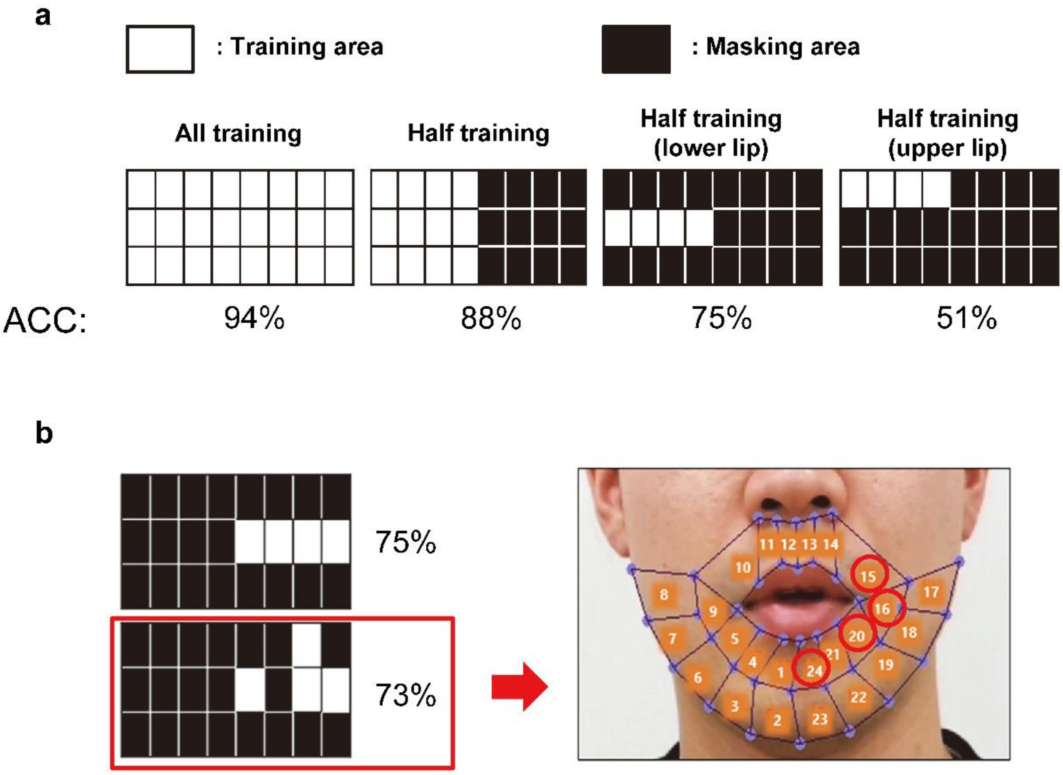

Supplementary Figure 2. Decision of sensor locations through area feature analysis (ablation study). a–b, Recognition accuracy of 26 NATO codes with some designated compartments masked**.**

This experiment evaluated the effectiveness of the sensor positioning. The dataset is classified into 26 NATO codes. A model based on a 3D convolutional network is used. When we trained using all the area features, the model's accuracy is 94%. When we use half of the area feature on the left side while masking the other half on the right side, the accuracy is 88%. This can be interpreted as having sufficient information with only half of the area feature because the whole muscles move symmetrically from side to side when we speak. When the performance of about four lower lip areas is compared with that of about four upper lip areas, the result of the former (75%) is better than that of the latter (51%). Additionally, we evaluated the performance with the area features of three lower lip areas and one upper lip area. Although the result (73%) is slightly lower than that of four lower lip areas (75%), a position shift of one sensor to an upper lip area enables a more convenient attachment of the sensors.

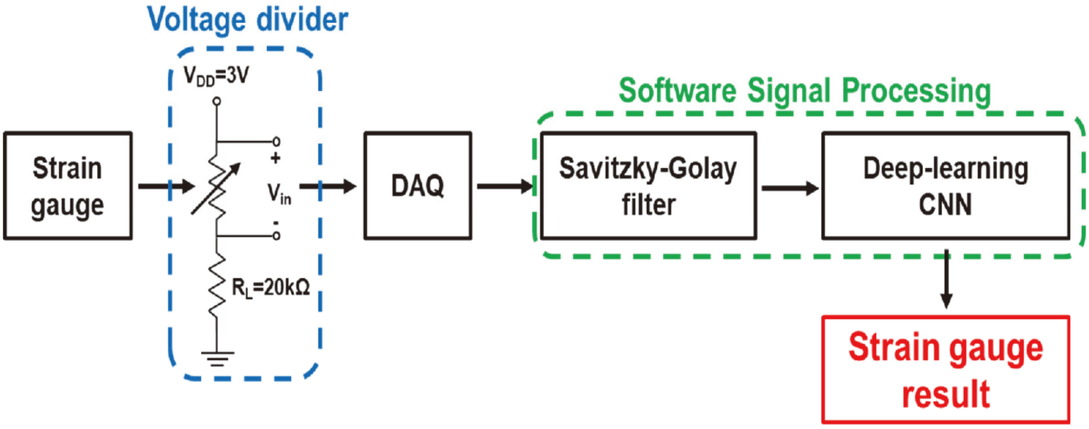

Supplementary Figure 3. Block diagram of sEMG data acquisition flow. The facial skin movements during silent speech are captured in the form of resistance change by epidermal strain sensors. The resistance change induces the voltage change by the voltage divider (V_s = 3V and R_L = 20 kΩ) and is monitored by the voltage input module of the DAQ system. The collected raw data are pre-processed with the Savitzky–Golay filter and converted to a 3D array format before being feature-extracted with a 3D convolutional neural network (CNN).

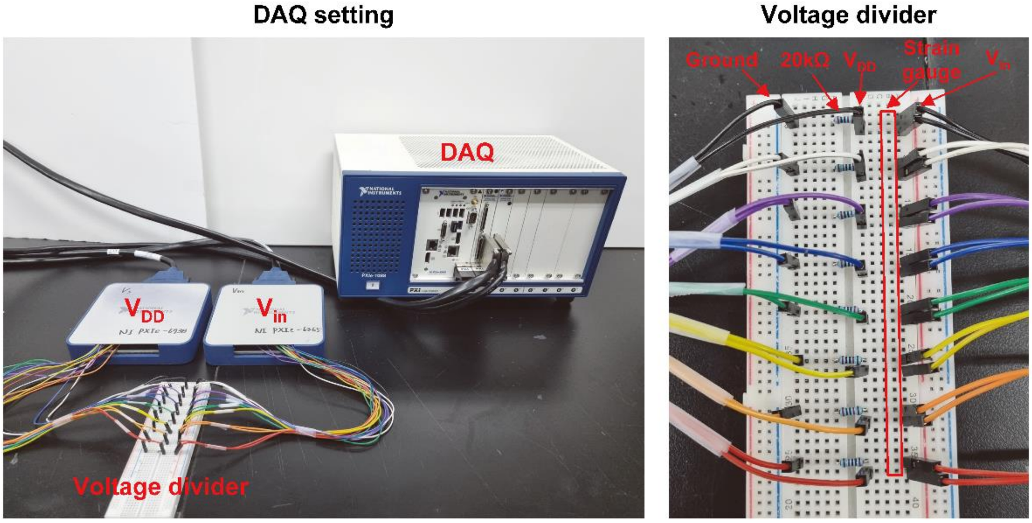

Supplementary Figure 4. Photograph of the strain data monitoring DAQ setup. The photograph consists of a voltage output module (NI PXIe-6738), voltage input module (NI PXIe-6365), voltage divider circuit, and an embedded controller (NI PXIe-8840) for measuring strain data with a frequency of 300 Hz.

Supplementary Note 1. Details in FEA simulation

The uniaxial stretching of the serpentine Si strain sensors in the mesh layout was simulated with the Piezoresistive Multiphysics model in COMSOL. Without loss of generality, the multilayered structures on the PDMS substrate (i.e., PI/Au/Cr/PI/PDMS and PI/Si/PI/PDMS) were modeled using a composite 2D shell with an effective Young's modulus of $E_{effective} = \sum E_i h_i / \sum h_i$, where E_i and h_i are the Young's modulus and thickness of each layer, respectively (Supplementary Table 1). The resistance change ($\Delta\rho$) of the piezoresistive sensor is related to the piezoresistive coefficient (π) and stress tensor (S) as $\Delta\rho = \pi \cdot S$. In the COMSOL simulation, the piezoresistive coefficient of p-type, single-crystal Si is given as

$$\pi = \begin{bmatrix} 6.6 & -1.1 & -1.1 & 0 & 0 & 0 \\ -1.1 & 6.6 & -1.1 & 0 & 0 & 0 \\ -1.1 & -1.1 & 6.6 & 0 & 0 & 0 \\ 0 & 0 & 0 & 138.1 & 0 & 0 \\ 0 & 0 & 0 & 0 & 138.1 & 0 \\ 0 & 0 & 0 & 0 & 0 & 138.1 \end{bmatrix} \cdot 10^{-10} \ (1/Pa).$$

As the electrical resistance can be easily obtained as the ratio of the applied electrical potential (e.g., 5 V) to the measured current, applying the tensile strain along the given direction yields a change of resistance in the Si strain sensor with 45° orientation[6, 7] upon stretching, which agrees reasonably well with the experimental measurements (Figs. 2d–e).

Supplementary Table 2. Material properties and thickness of each layer

Material names	Young's modulus (GPa)	Poisson's ratio	Thickness (μm)
**Polyimide**	4	0.34	6.8
**Au**	70	0.44	0.25
**Cr**	279	0.21	0.005
**P-type, single-crystalline Silicon**	166	0.27	0.3
**PDMS**	1.25×10^{-4}	0.49	500

Supplementary Table 3. Hyperparameters for 3D CNN-based deep-learning model

	**3D convolution based model**
**Learning rate**	0.0001
β_1	0.5
β_2	0.999
**Batch size**	100
**Epochs**	300

Supplementary Table 4. Statistics of strain datasets

	**Speaker A**		**Speaker B**	
	Label	# of datasets	Label	# of datasets
**Day 1**	A1	10	B1	15
**Day 2**	A2	9	B2	13
**Day 3**	A3	8	B3	14
**Day 4**	A4	5		
**Day 5**	A5	11		
**Day 6**	A6	15		
**Total**	**100**			

Supplementary Table 5. Comparison with the conventional methods

**Method**	**Strain Gauge**						**sEMG**
	**Fold 1**	**Fold 2**	**Fold 3**	**Fold 4**	**Fold 5**	**Avg**	**Val set**
PCA + LDA	6.4	8.85	16	10.15	11.7	10.62	2.2
PCA + SVM	11.5	15.65	46.85	31.15	22.25	25.48	2.55
SVM	58.8	71.3	76.25	72.7	67.1	69.23	4.9
CONV + SVM	79.95	84.8	90.2	90.55	85.85	86.27	40.85
Transformer	71.85	74.85	66.8	69.4	74.8	71.54	28.25
VGG	67.3	67.1	76.3	74.9	70.2	71.16	22.25
Ours	**80.1**	**87.85**	**91.55**	**90.5**	**87.65**	**87.53**	**42.60**

Supplementary Table 6. Recognition accuracy of all words (1–50) for 3 different classifier models.

Word	Accuracy (%)			Word	Accuracy (%)		
	Correlation	SVM	3D-Conv		Correlation	SVM	3D-Conv
ABSOLUTELY	21.05	85.00	100.00	ECONOMIC	21.05	85.00	100.00
ACCUSED	15.79	85.00	100.00	EMERGENCY	5.26	75.00	90.00
AFTERNOON	10.53	90.00	100.00	ENGLAND	5.26	55.00	90.00
AGREEMENT	5.26	75.00	100.00	EUROPE	15.79	90.00	95.00
ALLEGATIONS	10.53	75.00	100.00	EUROPEAN	10.53	80.00	90.00
ALMOST	5.26	60.00	90.00	EVERYBODY	5.26	70.00	90.00
AREAS	5.26	65.00	90.00	FAMILIES	0.00	55.00	75.00
AUTHORITIES	5.26	85.00	100.00	FAMILY	0.00	65.00	85.00
BECOME	36.84	95.00	90.00	FOLLOWING	5.26	60.00	90.00
BEFORE	31.58	65.00	80.00	FORMER	10.53	70.00	70.00
BEHIND	5.26	55.00	100.00	GERMANY	10.53	80.00	95.00
BELIEVE	0.00	45.00	70.00	GLOBAL	5.26	80.00	100.00
BENEFIT	5.26	55.00	85.00	HOMES	10.53	85.00	100.00
BETWEEN	15.79	85.00	95.00	HOSPITAL	10.53	95.00	95.00
CAMERON	10.53	90.00	100.00	HUNDREDS	0.00	70.00	80.00
CAMPAIGN	15.79	70.00	100.00	INCREASE	10.53	65.00	65.00
CHIEF	10.53	75.00	90.00	INFORMATION	10.53	85.00	95.00
COMMUNITY	15.79	80.00	95.00	INQUIRY	10.53	80.00	90.00
CONFLICT	5.26	70.00	95.00	INVESTMENT	21.05	85.00	90.00
CRIME	10.53	80.00	95.00	IRELAND	5.26	70.00	95.00
CUSTOMERS	15.79	85.00	100.00	ISLAMIC	10.53	85.00	90.00
DEGREES	15.79	45.00	85.00	ITSELF	15.79	95.00	100.00
DESCRIBED	10.53	80.00	90.00	LEADERSHIP	15.79	95.00	100.00
DESPITE	10.53	60.00	95.00	LEAVE	10.53	75.00	80.00
DETAILS	10.53	40.00	85.00	MAJORITY	0.00	25.00	90.00

Supplementary Table 7. Recognition accuracy of all words (51–100) for 3 different classifier models.

Word	Accuracy (%)			Word	Accuracy (%)		
	Correlation	SVM	3D-Conv		Correlation	SVM	3D-Conv
MEMBERS	10.53	75.00	85.00	QUESTIONS	0.00	75.00	95.00
MIGRANTS	10.53	70.00	80.00	RECORD	10.53	85.00	90.00
MOMENT	5.26	65.00	85.00	REFERENDUM	21.05	85.00	80.00
MORNING	15.79	85.00	100.00	REMEMBER	10.53	80.00	95.00
MOVING	10.53	80.00	90.00	REPORTS	10.53	60.00	85.00
NUMBERS	5.26	75.00	90.00	RESPONSE	5.26	90.00	95.00
OBAMA	10.53	90.00	90.00	SCOTLAND	10.53	80.00	95.00
OFFICERS	5.26	60.00	85.00	SECRETARY	5.26	90.00	100.00
OFFICIALS	10.53	70.00	100.00	SIGNIFICANT	5.26	90.00	100.00
OPERATION	10.53	70.00	85.00	SIMPLY	10.53	70.00	90.00
OPPOSITION	5.26	90.00	95.00	SMALL	21.05	90.00	100.00
PARLIAMENT	10.53	85.00	95.00	SUNSHINE	0.00	90.00	90.00
PARTS	15.79	60.00	85.00	TEMPERATURES	15.79	100.00	95.00
PATIENTS	5.26	55.00	80.00	THEMSELVES	10.53	70.00	95.00
PEOPLE	26.32	60.00	95.00	THOUSANDS	10.53	90.00	95.00
PERHAPS	5.26	65.00	90.00	TOMORROW	31.58	100.00	90.00
POLICY	10.53	60.00	95.00	VICTIMS	5.26	55.00	85.00
POLITICIANS	5.26	80.00	100.00	WEAPONS	5.26	75.00	100.00
POSSIBLE	0.00	60.00	85.00	WEEKEND	5.26	65.00	90.00
POTENTIAL	5.26	85.00	95.00	WELCOME	26.32	95.00	95.00
PRIME	5.26	85.00	90.00	WELFARE	10.53	100.00	100.00
PRIVATE	5.26	90.00	100.00	WESTERN	21.05	95.00	100.00
PROBLEMS	10.53	70.00	85.00	WESTMINSTER	10.53	100.00	90.00
PROCESS	5.26	75.00	90.00	WITHOUT	10.53	95.00	100.00
PROVIDE	5.26	85.00	100.00	WOMEN	5.26	90.00	80.00

Supplementary Table 8. Recognition accuracy change as training of unseen data increases 1.

	Training	Test	Accuracy(%)
From Scratch	A1	A6	6
	A1, A2	A6	7
	A1, A2, A3	A6	22
	A1, A2, A3, A4	A6	34
	A1, A2, A3, A4, A5	A6	35
	B + A1, A2, A3, A4, A5 ⋯⋯ (**)	A6	56
Transfer learning	(**) + 1 set among A6	A6	83
	(**) + 2 set among A6	A6	**88**

In this experiment, A6 datasets are fixed as test datasets, with two datasets chosen at random to be excluded for the transfer learning. Therefore, all of the training datasets are in a domain different from that of the test datasets (unseen data). When only A1 datasets are trained, the system classifies A6 datasets with very low accuracy due to the shortage of training datasets and the slight mismatch of sensor locations between the A1 and A6 datasets. However, this accuracy tends to gradually increase, even though the additional training datasets are all unseen data. Training B datasets from even different subjects increases the accuracy. It demonstrates that the low recognition rate of unseen data due to sensor or subject replacement can be gradually improved as the number of users of this system increases. Furthermore, the simple transfer learning of pre-excluded A6 datasets sharply increases the accuracy. Only two cycles of transfer learning increase the accuracy up to 88%, which enables the manageable customization for the initial use of the system.

Supplementary Table 9. Recognition accuracy change as training of unseen data increases 2.

	Training	Test	Accuracy(%)
From Scratch	B .... (*)	A6	16
	B + A1, A2	A6	25
	B + A1, A2	A6	38
	B + A1, A2, A3	A6	47
	B + A1, A2, A3, A4	A6	50
	B + A1, A2, A3, A4, A5 ...... (**)	A6	56
Transfer learning	(*) + 1 set among A6	A6	38
	(*) + 2 set among A6	A6	**68**
	(**) + 1 set among A6	A6	**83**
	(**) + 2 set among A6	A6	**88**

In this experiment, A6 datasets are fixed as test datasets, with two datasets chosen at random to be excluded for the transfer learning. Therefore, all of the training datasets are in a domain different from that of the test datasets (unseen data). When only B datasets are trained, the system classifies A6 datasets with very low accuracy due to the shortage of training datasets and the user dependency such as facial shapes or accents between the A and B datasets. However, this accuracy tends to gradually increase, even though the additional training datasets are all unseen data. It demonstrates that the low recognition rate of unseen data due to sensor or subject replacement can be gradually improved as the number of users of this system increases. Furthermore, the simple transfer learning of pre-excluded A6 datasets sharply increases the accuracy. Only two cycles of transfer learning increase the accuracy up to 88%, which enables the manageable customization for the initial use of the system.

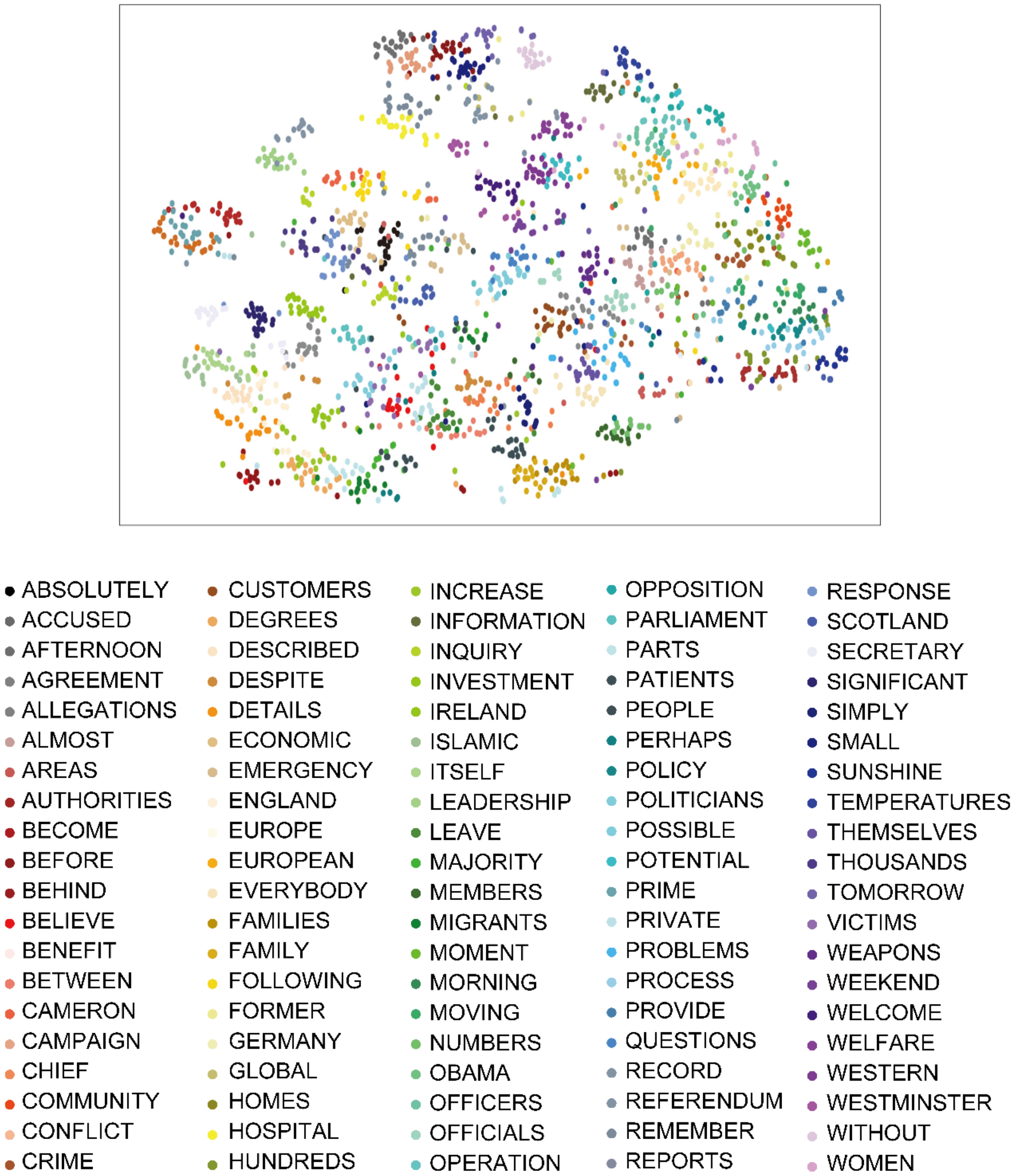

Supplementary Figure 5. Expanded view of t-SNE (strain). It shows the 100 words given in Fig. 4a in different colored points.

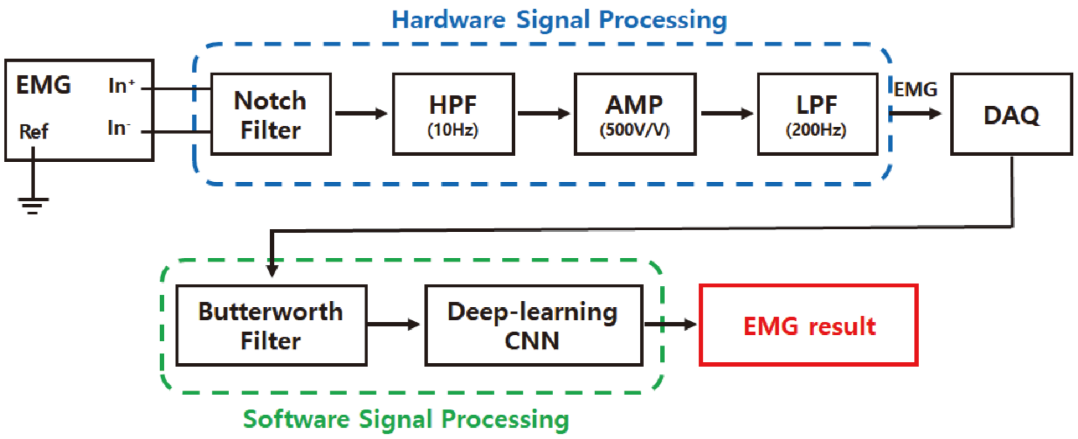

Supplementary Figure 6. Block diagram of sEMG data acquisition flow. The facial sEMG during silent speech is captured by epidermal EMG electrodes. This raw sEMG signal is pre-processed by a commercial EMG module (notch filter at 50 Hz, high-pass filter at 10 Hz, low-pass filter at 200 Hz, and amplifier at 500V/V), and collected by the voltage input module of the DAQ system. The resistance change induces the voltage change by the voltage divider (V_s = 3V and R_L = 20kΩ) and is monitored by the voltage input module of the DAQ system. The collected data is reprocessed using the Butterworth filter and converted to a 3D array format before being feature-extracted with a 3D CNN.

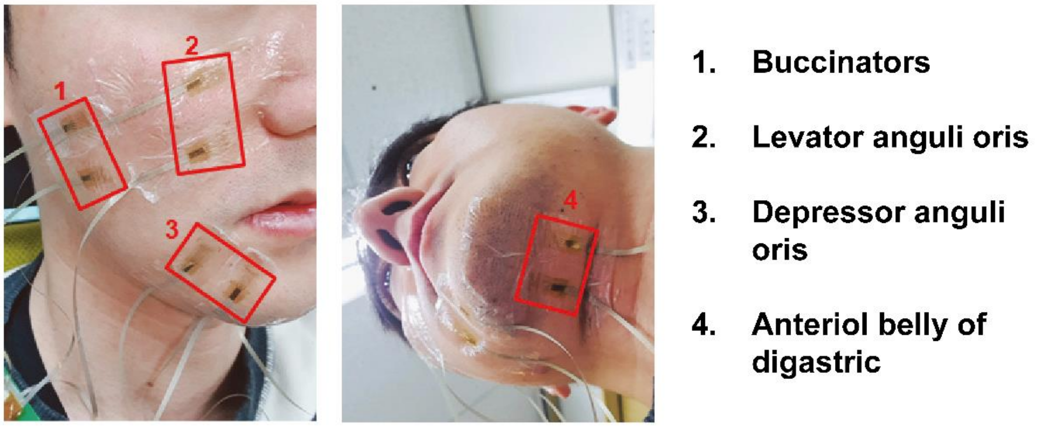

Supplementary Figure 7. Surface electrode attachment locations for monitoring EMG signals of facial muscles. Attachment locations 1–4 are buccinators, *levator anguli oris*, depressor *anguli oris*, and *anteriol* belly of digastric, respectively. Two electrodes are attached at a 2-cm interval in each location, and a common reference electrode is attached near the posterior mastoid.

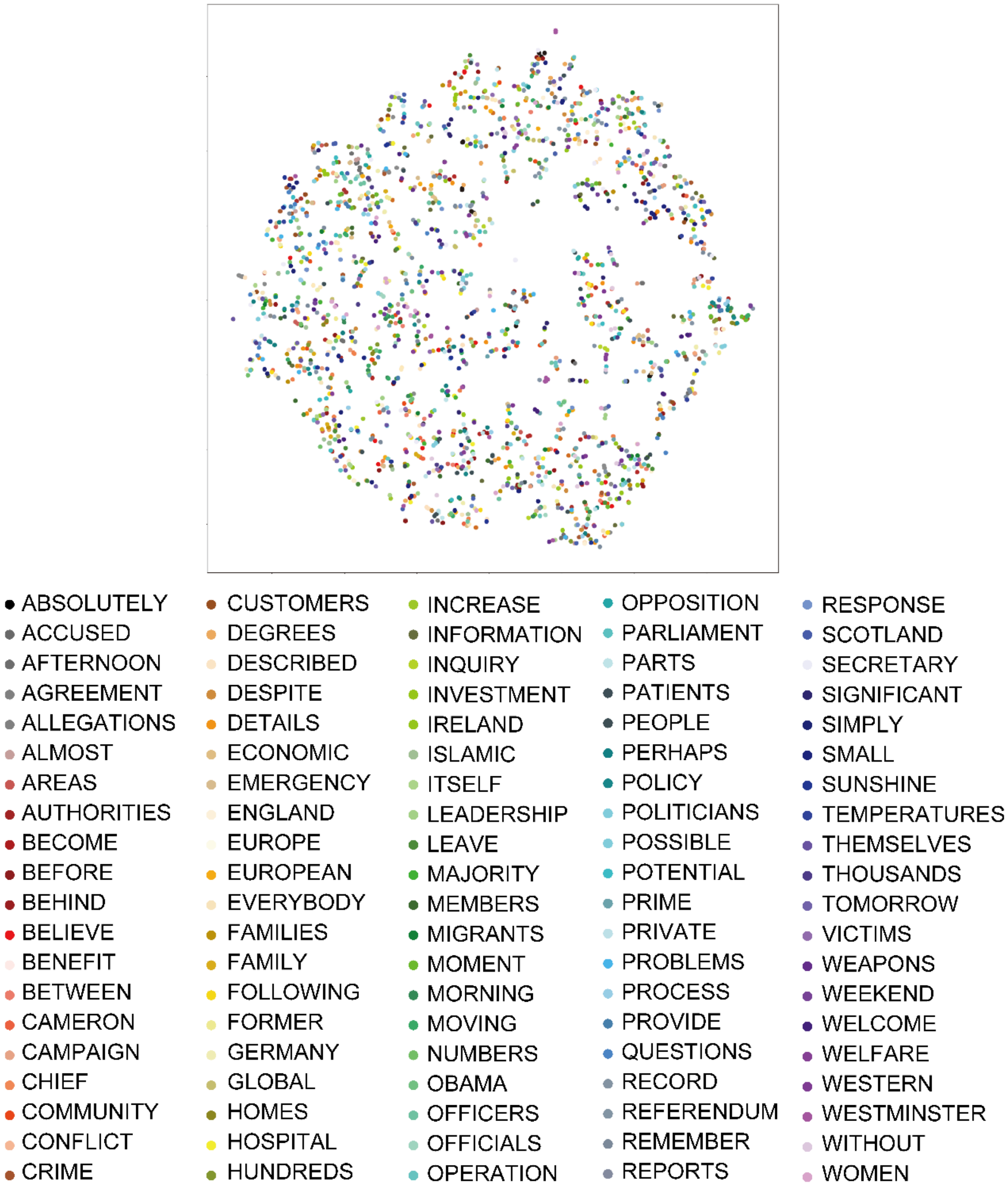

Supplementary Figure 8. Expanded view of t-SNE (sEMG). It shows the 100 words given in Fig. 5h in different colored points.

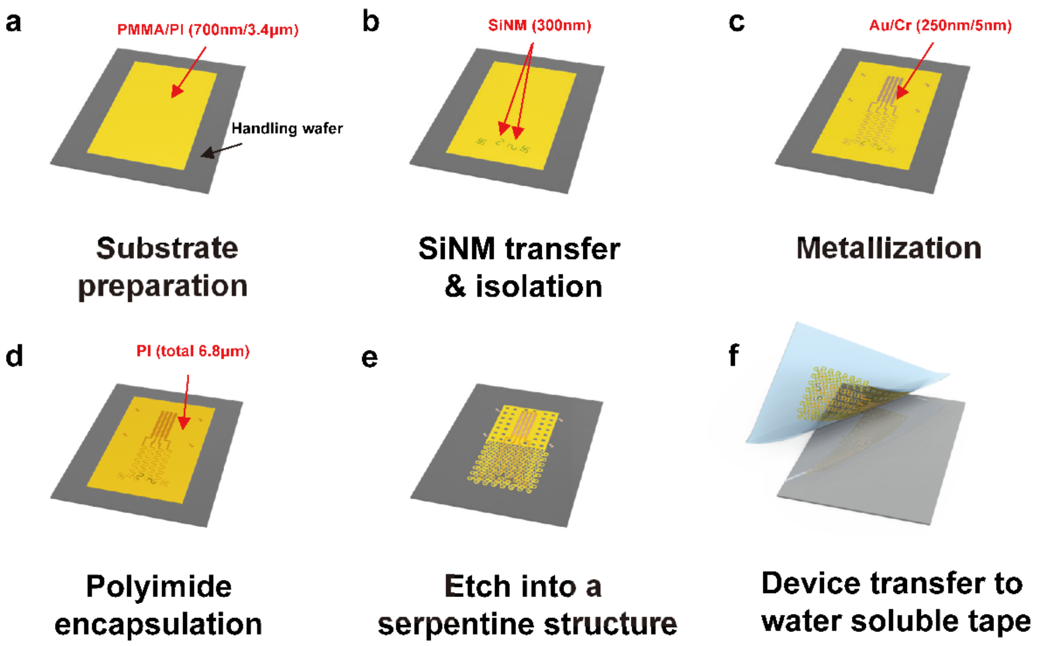

Supplementary Figure 9. Schematic diagram showing the fabrication procedures of the SiNM-based biaxial strain sensor. a, Spin coating PMMA and PI double-layer; **b,** Defining biaxial strain gauges by the transfer of SiNM using elastomer stamp and cell isolation; **c,** Metallization of Au/Cr interconnects by thermal evaporation; **d,** Device encapsulation with another PI double-layer; **e,** Device cutting using Cu mask and dry etching; **f,** Device release and transfer onto water-soluble tape.

Supplementary Note 2. Details of the fabrication steps to achieve a SiNM-based strain sensor.

SiNM doping

1. Surface cleaning of SOI chips (Device layer of 300 nm, BOX layer of 1 μm, handling wafer of 730 μm) using piranha solution (3:1) at 100°C for 15 min;
2. BOE (6:1) to remove native/chemical oxide for 5 s;
3. Dope the entire area with boron by ion implantation (power of 30 keV, dose of 5×10^{14} $cm^{-2)}$;
4. Rapid thermal annealing at 1050°C for 90 s.

Microhole

5. Piranha and BOE cleaning;
6. UV-lithography to define microhole array (diameter of 3 μm, pitch of 50 μm) using positive PR (MICROPOSIT S1805) and developer (AZ 300 mif);
7. Dry etch of Si by RIE (Torr of 150 m, SF6 of 40 sccm, power of 150 W for 50 s).

Substrate preparation

8. Piranha cleaning of thermal oxide wafer (SiO_2 of 500 μm);

9. Spin coat PMMA A8 (500 rpm for 10 s, 1000 rpm for 35 s); Soft bake at 110°C for 1 min; Cure at 180°C for 3 min;
10. Spin coat PI (500 rpm for 10 s, 3000 rpm for 30 s); Soft bake at 110°C for 3 min and then at 150°C for 3 min; Cure at 210°C for 120 min.

Transfer printing

11. Cure PDMS stamp (4:1) at 40°C for 24 h;
12. Spin coat the second PI on the above substrate (500 rpm for 10 s, 3000 rpm for 30 s); Soft bake at 110°C for 40 s;
13. HF wet etch BOX layer of SOI chips for 25 min;
14. Water rinsing of SOI chips;
15. Transfer of the device layer of SOI chips onto the PDMS stamp; Press the stamp onto the substrate;
16. Bake at 110°C for 40 s;
17. Lift off PDMS stamp;
18. Bake at 150°C for 3 min;
19. PR removal using acetone, IPA, and DI water;
20. Cure at 210°C for 120 min.

Strain gauge isolation

21. UV-lithography to define biaxial strain gauges using positive PR (MICROPOSIT S1805) and developer (AZ 300 mif);
22. Dry etch of Si by RIE (Torr of 150 m, SF6 of 40 sccm, power of 150 W for 50 s);
23. PR removal using acetone, IPA, and DI water.

Metallization

24. BOE (6:1) cleaning for 5 s to remove native oxide;
25. Deposit Au/Cr, 250 nm/5 nm by thermal evaporation;
26. UV-lithography to define metal interconnects using positive PR (AZ 5214E) and developer (AZ 300 mif);
27. Wet etch using Au etchant for 15 s; Cr etchant for 10 s;
28. PR removal using acetone, IPA, and DI water.

Encapsulation

29. Spin coat the third PI (500 rpm for 10 s, 3000 rpm for 30 s); Soft bake at 110°C for 3 min and then at 150°C for 3 min;
30. Spin coat the fourth PI (500 rpm for 10 s, 3000 rpm for 30 s); Soft bake at 110°C for 3 min and then at 150°C for 3 min;

31. Cure at 210°C for 120 min.

Patterning into serpentine design

32. Deposit Cu, 150 nm by thermal evaporation;
33. UV-lithography to define metal masks using positive PR (AZ 5214E) and developer (AZ 300 mif);
34. PR removal using acetone, IPA, and DI water;
35. Dry etch of PI by RIE (Torr of 390 m, SF6 of 100 sccm, power of 200 W for 30 min);
36. Wet etch of metal masks using Cu etchant.

Transfer to water-soluble tape

37. Dissolve the PMMA layer by immersing in acetone at 100°C for 10 min;
38. Solder with ACF cable;
39. Attach water-soluble tape, and lift off the release device.

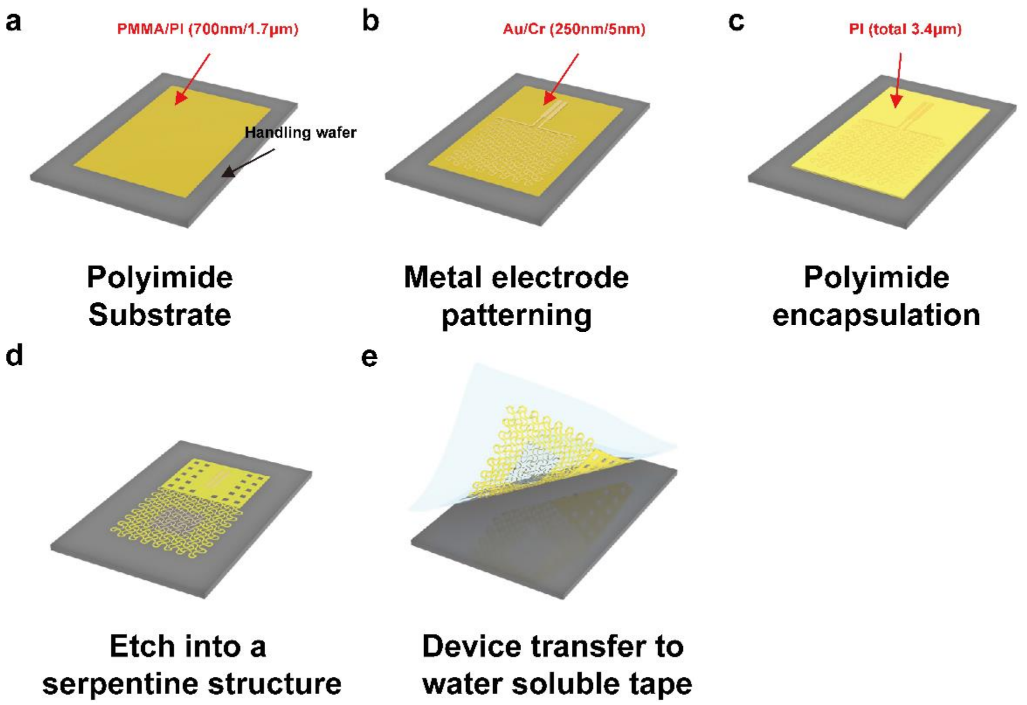

Supplementary Figure 10. Schematic diagram showing the fabrication procedures of the sEMG electrode. a, Spin coating PMMA and PI layer; **b,** Defining metal electrode and interconnects; **c,** Device encapsulation with another PI layer; **d,** Device cutting and through-opening using Cu mask and dry etching; **e,** Device release and transfer onto water-soluble tape.

Supplementary Note 3. Details of the fabrication steps to achieve a stretchable sEMG sensor.

Substrate preparation

1. Piranha cleaning of thermal oxide wafer (SiO_2 of 500 μm);
2. Spin coat PMMA A8 (500 rpm for 10 s, 1000 rpm for 35 s); Soft bake at 110°C for 1 min; Cure at 180°C for 3 min;
3. Spin coat PI (500 rpm for 10 s, 3000 rpm for 30 s); Soft bake at 110°C for 3 min and then at 150°C for 3 min; Cure at 210°C for 120 min.

Metal electrode and interconnect define

4. Deposit Au/Cr, 160 nm/5 nm by thermal evaporation;
5. UV-lithography to define metal electrodes and interconnects using positive PR (AZ 5214E) and developer (AZ 300 mif);
6. Wet etch using Au etchant for 15 s; Cr etchant for 10 s;
7. PR removal using acetone, IPA, and DI water.

Encapsulation

8. Spin coat the second PI (500 rpm for 10 s, 3000 rpm for 30 s); Soft bake at 110°C for 3 min and then at 150°C for 3 min; Cure at 210°C for 120 min.

Electrode via opening and patterning into serpentine design

9. Deposit Cu, 150 nm by thermal evaporation;
10. UV-lithography to define metal masks using positive PR (AZ 5214E) and developer (AZ 300 mif);
11. PR removal using acetone, IPA, and DI water;
12. Dry etch of PI by RIE (Torr of 390 m, SF6 of 100 sccm, power of 200 W for 15 min);
13. Wet etch of metal masks using Cu etchant.

Transfer to water-soluble tape

14. Dissolve the PMMA layer by immersing in acetone at 100°C for 10 min;
15. Solder with ACF cable;
16. Attach water-soluble tape, and lift off the release device.

Supplementary Table 10. Model details for SiNM-based Strain Gauge

Layer name	Operator	Kernel size	Padding	Stride	Channel size	Ouput Size
Conv 1	$\begin{bmatrix} Conv3d \\ InstanceNorm3d \\ ReLU \\ Dropout3d\ (0.3) \end{bmatrix}$	3 x 3 x 3	(1, 1, 1)	(1, 1, 2)	32	2 x 4 x 300
Conv 2	$\begin{bmatrix} Conv3d \\ InstanceNorm3d \\ ReLU \\ Dropout3d\ (0.3) \end{bmatrix}$	3 x 3 x 3	(1, 1, 1)	(1, 1, 2)	32	2 x 4 x 150
	$\begin{bmatrix} Conv3d \\ InstanceNorm3d \\ ReLU \\ Dropout3d\ (0.3) \end{bmatrix}$	3 x 3 x 3	(1, 1, 1)	(1, 1, 1)		
Conv 3	$\begin{bmatrix} Conv3d \\ InstanceNorm3d \end{bmatrix}$	1 x 3 x 3	(0, 1, 1)	(1, 2, 2)	64	2 x 2 x 75
Conv 4	$\begin{bmatrix} Conv3d \\ InstanceNorm3d \\ ReLU \\ Dropout3d\ (0.3) \end{bmatrix}$	3 x 3 x 3	(1, 1, 1)	(1, 1, 2)	64	2 x 2 x 38
	$\begin{bmatrix} Conv3d \\ InstanceNorm3d \\ ReLU \\ Dropout3d\ (0.3) \end{bmatrix}$	3 x 3 x 3	(1, 1, 1)	(1, 1, 1)		
Conv 5	$\begin{bmatrix} Conv3d \\ InstanceNorm3d \end{bmatrix}$	3 x 3 x 3	(1, 1, 1)	(1, 2, 2)	128	2 x 1 x 19
Conv 6	$\begin{bmatrix} Conv3d \\ InstanceNorm3d \\ ReLU \\ Dropout3d\ (0.3) \end{bmatrix}$	3 x 3 x 3	(1, 1, 1)	(1, 1, 2)	128	2 x 1 x 10
	$\begin{bmatrix} Conv3d \\ InstanceNorm3d \\ ReLU \\ Dropout3d\ (0.3) \end{bmatrix}$	3 x 3 x 3	(1, 1, 1)	(1, 1, 1)		
Conv 7	$\begin{bmatrix} Conv3d \\ InstanceNorm3d \end{bmatrix}$	3 x 3 x 3	(1, 1, 1)	(1, 2, 2)	256	2 x 1 x 5
Conv 8	$\begin{bmatrix} Conv3d \\ InstanceNorm3d \\ ReLU \\ Dropout3d\ (0.3) \end{bmatrix}$	3 x 3 x 3	(1, 1, 1)	(1, 1, 2)	256	2 x 1 x 3
	$\begin{bmatrix} Conv3d \\ InstanceNorm3d \\ ReLU \\ Dropout3d\ (0.3) \end{bmatrix}$	3 x 3 x 3	(1, 1, 1)	(1, 1, 1)		
FC 1	Linear	-				512

Supplementary Table 11. Model details for sEMG

Layer name	Operator	Kernel size	Padding	Stride	Channel size	Output Size
Conv 1	$\begin{bmatrix} Conv3d \\ InstanceNorm3d \\ ReLU \\ Dropout3d\ (0.3) \end{bmatrix}$	3 x 3 x 3	(1, 1, 1)	(1, 1, 2)	32	1 x 4 x 1000
Conv 2	$\begin{bmatrix} Conv3d \\ InstanceNorm3d \\ ReLU \\ Dropout3d\ (0.3) \end{bmatrix}$	3 x 3 x 3	(1, 1, 1)	(1, 1, 2)	32	1 x 4 x 500
	$\begin{bmatrix} Conv3d \\ InstanceNorm3d \\ ReLU \\ Dropout3d\ (0.3) \end{bmatrix}$	3 x 3 x 3	(1, 1, 1)	(1, 1, 1)		
Conv 3	$\begin{bmatrix} Conv3d \\ InstanceNorm3d \end{bmatrix}$	1 x 3 x 3	(0, 1, 1)	(1, 2, 2)	64	1 x 2 x 250
Conv 4	$\begin{bmatrix} Conv3d \\ InstanceNorm3d \\ ReLU \\ Dropout3d\ (0.3) \end{bmatrix}$	3 x 3 x 3	(1, 1, 1)	(1, 1, 2)	64	1 x 2 x 125
	$\begin{bmatrix} Conv3d \\ InstanceNorm3d \\ ReLU \\ Dropout3d\ (0.3) \end{bmatrix}$	3 x 3 x 3	(1, 1, 1)	(1, 1, 1)		
Conv 5	$\begin{bmatrix} Conv3d \\ InstanceNorm3d \end{bmatrix}$	3 x 3 x 3	(1, 1, 1)	(1, 2, 2)	128	1 x 1 x 63
Conv 6	$\begin{bmatrix} Conv3d \\ InstanceNorm3d \\ ReLU \\ Dropout3d\ (0.3) \end{bmatrix}$	3 x 3 x 3	(1, 1, 1)	(1, 1, 2)	128	1 x 1 x 32
	$\begin{bmatrix} Conv3d \\ InstanceNorm3d \\ ReLU \\ Dropout3d\ (0.3) \end{bmatrix}$	3 x 3 x 3	(1, 1, 1)	(1, 1, 1)		
Conv 7	$\begin{bmatrix} Conv3d \\ InstanceNorm3d \end{bmatrix}$	3 x 3 x 3	(1, 1, 1)	(1, 2, 2)	256	1 x 1 x 16
Conv 8	$\begin{bmatrix} Conv3d \\ InstanceNorm3d \\ ReLU \\ Dropout3d\ (0.3) \end{bmatrix}$	3 x 3 x 3	(1, 1, 1)	(1, 1, 2)	256	1 x 1 x 8
	$\begin{bmatrix} Conv3d \\ InstanceNorm3d \\ ReLU \\ Dropout3d\ (0.3) \end{bmatrix}$	3 x 3 x 3	(1, 1, 1)	(1, 1, 1)		
FC 1	Linear	-				512

Supplementary Table 12. Detailed structure of Transformer

Layer name	Operator	Kernel Size	Padding	Stride	Channel Size	Output Size
Conv 1	$\begin{bmatrix} Conv1d \\ ReLU \\ Batchnorm \end{bmatrix}$	5	2	2	32	300
Conv 2	$\begin{bmatrix} Conv1d \\ ReLU \\ Dropout\ (0.2) \end{bmatrix}$	5	2	2	64	150
Conv 3	$\begin{bmatrix} Conv1d \\ ReLU \\ Batchnorm \end{bmatrix}$	5	2	2	128	75
Conv 4	$\begin{bmatrix} Conv1d \\ ReLU \\ Dropout\ (0.2) \end{bmatrix}$	5	2	2	256	38
Transformer encoder 1	-				256	38
Transformer encoder 2	-				256	38
Self-attention pooling						256
FC 1	$\begin{bmatrix} Linear \\ ReLU \end{bmatrix}$	-				400
FC 2	$\begin{bmatrix} Linear \\ ReLU \end{bmatrix}$	-				400
FC 3	$\begin{bmatrix} Linear \\ ReLU \end{bmatrix}$	-				400
FC 4	Linear	-				100

Supplementary Table 13. Detailed structure of VGGNet

Layer name	Operator	Kernel Size	Padding	Stride	Channel Size	Output Size
Conv 1	Conv2d	(3, 7)	(1, 3)	(1, 2)	64	8 x 300
Conv 2	$\begin{bmatrix} Conv2d \\ Batchnorm \\ ReLU \\ Maxpool \end{bmatrix}$	(3, 3)	(1, 1)	(1, 1)	64	4 x 150
Conv 3	$\begin{bmatrix} Conv2d \\ Batchnorm \\ ReLU \\ Maxpool \end{bmatrix}$	(3, 3)	(1, 1)	(1, 1)	128	2 x 75
Conv 4	$\begin{bmatrix} Conv2d \\ Batchnorm \\ ReLU \end{bmatrix}$	(3, 3)	(1, 1)	(1, 1)	256	1 x 37
	$\begin{bmatrix} Conv2d \\ Batchnorm \\ ReLU \\ Maxpool \end{bmatrix}$	(3, 3)	(1, 1)	(1, 1)	256	
Conv 5	$\begin{bmatrix} Conv2d \\ Batchnorm \\ ReLU \end{bmatrix}$	(1, 3)	(0, 1)	(1, 1)	512	1 x 18
	$\begin{bmatrix} Conv2d \\ Batchnorm \\ ReLU \\ Maxpool \end{bmatrix}$	(1, 3)	(0, 1)	(1, 1)	512	
Conv 6	$\begin{bmatrix} Conv2d \\ Batchnorm \\ ReLU \end{bmatrix}$	(1, 3)	(0, 1)	(1, 1)	512	1 x 9
	$\begin{bmatrix} Conv2d \\ Batchnorm \\ ReLU \\ Maxpool \end{bmatrix}$	(1, 3)	(0, 1)	(1, 1)	512	
Statistic pooling						1024
FC 1	$\begin{bmatrix} Linear \\ ReLU \\ Dropout(0.65) \end{bmatrix}$	-				4096
FC 2	$\begin{bmatrix} Linear \\ ReLU \\ Dropout(0.65) \end{bmatrix}$	-				4096
FC 3	Linear	-				100

Supplementary References

1. Ravenscroft, D. et al. Machine learning methods for automatic silent speech recognition using a wearable graphene strain gauge sensor. *Sensors* **22**, 299 (2021).
2. Wang, Y. et al. A durable nanomesh on-skin strain gauge for natural skin motion monitoring

with minimum mechanical constraints. *Science Advances* **6**, eabb7043 (2020).
3. Song, X. et al. A graphene-coated silk-spandex fabric strain sensor for human movement monitoring and recognition. *Nanotechnology* **32**, 215501 (2021).
4. Wang, M. et al. Gesture recognition using a bioinspired learning architecture that integrates visual data with somatosensory data from stretchable sensors. *Nature Electronics* **3**, 563-570 (2020).
5. Li, L., Jiang, S., Shull, P.B. & Gu, G. SkinGest: artificial skin for gesture recognition via filmy stretchable strain sensors. *Advanced Robotics* **32**, 1112-1121 (2018).
6. Basov, M. & Prigodskiy, D. Modeling of sensitive element for pressure sensor based on bipolar piezotransistor. (2021).
7. Romdhane, M.C.B., Mrad, H., Erchiqui, F. & Mrad, R.B. in IOP Conference Series: Materials Science and Engineering, Vol. 521 012004 (IOP Publishing, 2019).
