# Supplementary material for: Ultrathin crystalline-silicon-based strain gauges with deep learning algorithms for silent speech interfaces
Source: Nat Commun. 2022 Oct 3;13:5815. doi: 10.1038/s41467-022-33457-9 (PMC9530138; doi:10.1038/s41467-022-33457-9)
Supplement: Supplementary file 3 — Description of Additional Supplementary Files [file 41467_2022_33457_MOESM3_ESM.docx]

File Name: Supplementary Movie 1

Description: Demonstration of word recognition (speaker A)

File Name: Supplementary Movie 2

Description: Demonstration of word recognition (speaker B)

File Name: Supplementary Movie 3

Description: Demonstration of sensor attachment process
